# A168 RATES OF *CLOSTRIDIOIDES DIFFICILE* INFECTIONS REQUIRING HOSPITALIZATION DURING THE COVID-19 PANDEMIC

**DOI:** 10.1093/jcag/gwad061.168

**Published:** 2024-02-14

**Authors:** J Besney, K Buhler, G G Kaplan, C Lu, C Seow, K Novak, R Panaccione, C Ma

**Affiliations:** University of Calgary, Calgary, AB, Canada; University of Calgary, Calgary, AB, Canada; University of Calgary, Calgary, AB, Canada; University of Calgary, Calgary, AB, Canada; University of Calgary, Calgary, AB, Canada; University of Calgary, Calgary, AB, Canada; University of Calgary, Calgary, AB, Canada; University of Calgary, Calgary, AB, Canada

## Abstract

**Background:**

*Clostridium difficile* infection (CDI) is the most common cause of nosocomial infectious diarrhea and is associated with a substantial burden of morbidity and mortality. Most cases of CDI are acquired through health care setting contacts, where appropriate infection prevention and control (IPC) measures such as contact isolation and proper hand hygiene have been demonstrated to prevent horizontal transmission.

**Aims:**

We aimed to evaluate whether there was a reduction in CDI-related hospitalizations during the COVID-19 pandemic when an increased emphasis was placed on maintaining IPC measures.

**Methods:**

We analyzed data from the National Inpatient Sample (NIS) between January 2018 and November 2020. The NIS is the largest publicly available administrative health database in the United States, capturing ampersand:003E7 million hospital admissions annually. All analyses were weighted for the complex survey design of the NIS. Temporal trends in monthly admissions for patients with CDI defined using International Classification of Disease 10^th^ revision codes and excluding patients with established CDI carrier status were evaluated using joinpoint regression. Changes over time were expressed as monthly percent change (MPC). To establish whether temporal changes were related to a reduction in CDI or whether these were more generalized effects due to the pandemic, we compared these temporal trends to rates of hospitalizations for inflammatory bowel disease (IBD) and stroke.

**Results:**

A total of 1,501,415 CDI admissions were identified. The mean age was 64.6 years and 54.2% of patient’s were female. 4.5% of admissions for CDI were associated with in-hospital mortality. Trends in hospitalization are summarized in **Figure 1**. Compared to the pre-pandemic period (January 2018-December 2019) CDI-related hospitalizations decreased by 9.1%/month [95% CI: -19.2%, +2.0%] (p=0.12) in early 2020 (January-March 2020). This reduction was not sustained: hospitalizations subsequently increased by +8.0% [95% CI: -4.0%, +21.6%] (p=0.19) in mid-2020 (April-June 2020) and were stable after July 2020 (MPC -0.9% [95% CI: -3.5%, +1.7%], p=0.48). Similar patterns were observed for both IBD and stroke-related hospitalizations. Total hospitalizations per month for CDI were similar by June 2020 as compared to the year preceding the pandemic.

**Conclusions:**

Although an initial decrease in the rates of CDI hospitalizations were seen early in the pandemic (January-March 2020), this pattern was similarly seen for other chronic and acute conditions, and this has not been sustained long-term.

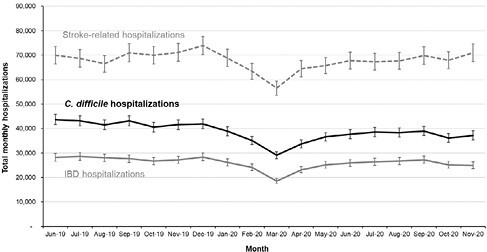

**Figure 1**. Rates of CDI admissions (2018-2020)

**Funding Agencies:**

None

